# A Rare Presentation of Narcolepsy With Cataplexy After Vaccines in a Genetically Susceptible Elderly Woman: A Case Report

**DOI:** 10.7759/cureus.40997

**Published:** 2023-06-26

**Authors:** Ram K Verma, Vinita Prasad, Subhendu Rath, Varun Monga, Gagandeep Dhillon

**Affiliations:** 1 Sleep Medicine, Parkview Health System, Fort Wayne, USA; 2 Psychiatry, Parkview Health System, Fort Wayne, USA; 3 Department of Neurology, Virginia Commonwealth University School of Medicine, Richmond, USA; 4 Psychiatry, Banner Health, Phoenix, USA; 5 Internal Medicine, Baltimore Washington Medical Center (BWMC), Glen Burnie, USA

**Keywords:** genetically, elderly, vaccines, cataplexy, narcolepsy

## Abstract

Observing cataplexy episodes during an office visit is extremely rare as they are usually triggered by laughter or emotional stress. Narcolepsy usually occurs in the younger population. We report a case of a 65-year-old Caucasian female with a past medical history of obesity who developed excessive daytime sleepiness, fatigue, and sleep attacks five weeks after getting influenza and pneumococcal vaccines. The presentation of cataplexy was atypical. Several episodes of cataplexy were observed during the office visit without any emotional trigger. Further workup, including polysomnography (PSG), was positive for obstructive sleep apnea, controlled with continuous positive airway pressure (CPAP) use. Later, she had PSG with CPAP use, which optimally controlled obstructive sleep apnea, followed by multiple sleep latency tests (MSLT) with CPAP use. It was positive for narcolepsy with a mean sleep latency of 1.6 minutes with sleep onset rapid eye movement (REM) in five out of five naps. Her cerebrospinal fluid (CSF) hypocretin level was extremely low at 50 pg/ml, usually seen in narcolepsy with cataplexy. She was also positive for human leukocyte antigen (HLA) DBQ1*06:02. The diagnosis of narcolepsy with cataplexy was made, which improved with medications for narcolepsy.

## Introduction

Narcolepsy, a chronic neurological disorder, is characterized by excessive daytime sleepiness, loss of muscle tone (cataplexy), sleep paralysis, and hypnopompic/hypnagogic hallucinations. Other features required to diagnose narcolepsy are cerebrospinal fluid (CSF) hypocretin deficiency and mean sleep latency of less than eight minutes with two sleep onset rapid eye movements (REMs). REM sleep latency less than or equal to 15 minutes during overnight polysomnography (PSG) can be considered one sleep onset REM [1]. The prevalence of narcolepsy varies across populations and geographic regions. A study looking at approximately 8.4 million patients using Truven Health MarketScan Commercial Dissertation Database (THMCDD) found a prevalence (per 100,000 patients) of narcolepsy at 79.4, 65.4 without cataplexy and 14.0 with cataplexy [2]. Although a rare disorder, narcolepsy can substantially negatively impact mental health. It can cause economic burdens such as work impairment and health-related decreased quality of life [3]. Narcolepsy usually occurs in the second decade of life, with another peak in the fourth decade [4]. Narcolepsy with cataplexy is considered to have autoimmune pathology, which may result in the disappearance of hypocretin neurons [1]. Influenza vaccination increases the risk of narcolepsy by two- to seven-fold in adults and five- to 14-fold in children and adolescents as per a metanalysis which mainly included the European population, and it has not been studied widely in different ethnicity [5]. About 72-93% of human leukocyte antigen (HLA) DBQ1*06:02 narcolepsy and cataplexy patients have low hypocretin levels [6-7]. About 25% of the general European population is positive for HLA DQB1*06:02 [8,9].

In this case report, we discuss an incredibly challenging diagnostic dilemma: a genetically susceptible older woman presented after influenza and pneumococcal vaccination with severe cataplexy not associated with significant emotional triggers.

## Case presentation

A 65-year-old Caucasian female with a past medical history of obesity with no other medication than baby aspirin was referred to a sleep medicine clinic for evaluation of new-onset fatigue, sleepiness, episodic weakness, and sleep attacks. The patient had flu and pneumococcal vaccines about five to six weeks before developing excessive daytime sleepiness, fatigue, and episodic weakness with sleep attacks. She did not have excessive daytime sleepiness or fatigue before. There was no significant change in her weight. She was also hospitalized for a workup for fatigue and weakness, where she was witnessed to have several "episodes" in which she would appear to fall asleep mid-conversation suddenly and her lips would quiver. In addition, her speech would slur until she could no longer form words. These episodes would last several seconds, and then the patient would return to her baseline with no neuro deficits. She reported continued awareness and no loss of consciousness during the episodes but could not speak or move her arms and legs. The patient had a computed tomography (CT) head and electroencephalography (EEG), which were unremarkable. She also had lab work including thyroid stimulating hormone (TSH), magnesium, and erythrocyte sedimentation rate (ESR), which were normal. She also had an MRI brain, which showed small vessel ischemic changes. She also had carotid Doppler and transthoracic echocardiography, which was unremarkable. She also complained of mild snoring but no excessive daytime somnolence prior to vaccination. Cataplexy episodes are exceedingly rare to be observed during an office visit. The patient also reported that her episodes also occur in the presence of unfamiliar persons. This patient had a couple of episodes of weakness of extremities, slowing of voice, and head limps with no fall during the office visit in the absence of any laughter. The patient did not fall down and regained her strength after 30-60 seconds. She also complained of hypnagogic hallucinations but denied any hypnopompic hallucinations or sleep paralysis. On physical examination, the patient had a blood pressure of 138/82 mmHg, pulse rate 93 per minute, respiratory rate 15/min, oxygen saturation (SpO2) 97%, neck size 15 inches, and body mass index (BMI) 35.6 kg/m2. Her Epworth Sleepiness scale score was 20/24, which indicates excessive daytime sleepiness. During the office visit, three to five similar episodes of cataplexy were noted. The patient was slightly frustrated due to her repetitive episodes of sleep attacks and fatigue.

**Figure 1:**
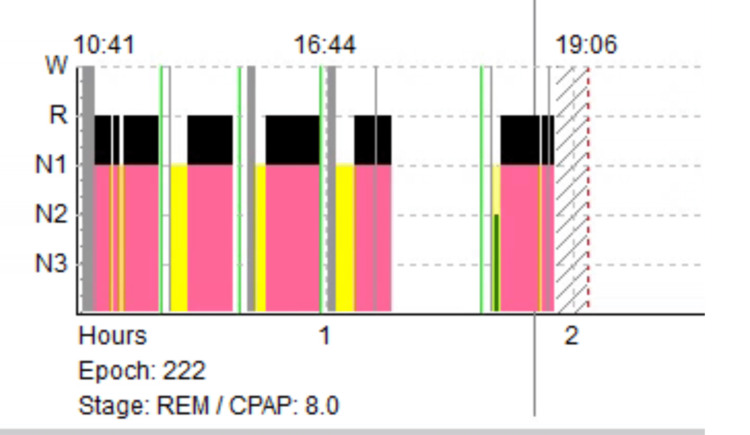
MSLT hypnogram Different stages of sleep: Wake (W), Light sleep (N1 and N2), deep sleep (N3), and REM (R) sleep. The patient had REM sleep in every nap. CPAP of 8 cm of water was also used during MSLT naps to control underlying obstructive sleep apnea. MSLT: Multiple Sleep Latency Test, REM: Rapid Eye Movement, CPAP: Continuous Positive Airway Pressure

Otherwise, the rest of the examination was unremarkable. The patient was seen by a neurologist while inpatient prior to an office visit and was suspected of having narcolepsy. The patient was seen by another neurologist as an outpatient for a second opinion, given an atypical presentation to rule out a differential diagnosis of myasthenia gravis. She also had repetitive electromyography, which came normal. She also had antibodies tests such as acetylcholine antibody, muscle-specific kinase antibody (anti-MuSK), and low-density lipoprotein receptor-related protein 4 antibody (LRP-4), which were negative. She was also found to be negative for coronavirus disease 2019 (COVID-19). She was eventually diagnosed with conversion disorder. She received cognitive behavior therapy, but it did not help much. Then, the patient was seen in the sleep clinic. The patient never had sleep studies before. Given her high BMI and snoring history, there was a possibility of undiagnosed sleep apnea as well. The patient was ordered PSG, followed by multiple sleep latency tests (MSLT). The patient had overnight PSG and was found to have obstructive sleep apnea with an overall apnea-hypopnea index (AHI) of 8.5 per hour with REM AHI of 30.9 per hour with the lowest oxygen saturation of 86%. After finding sleep apnea during PSG, the decision was made to control sleep apnea first and then repeat PSG with continuous positive airway pressure (CPAP) followed by MSLT later. The patient was put on Auto CPAP of 6-20 cm of water. With CPAP use, the patient noticed an improvement in the quality of sleep. She was compliant with CPAP use. Her average CPAP pressure was around 8 cm of water while on auto CPAP. Despite CPAP use, the patient remained symptomatic with fatigue, sleepiness, and cataplexy, so she was put on armodafinil initially and later switched to Adderall and protriptyline, given suboptimal benefit. The patient was also referred to the tertiary care center given the unusual presentation of cataplexy, where she had PSG with CPAP of 8 cm of water. The PSG with CPAP use revealed normalization of sleep apnea with overall AHI of 0.6 per hour with a sleep latency of 4.5 minutes and REM latency of eight minutes with a total sleep time of 505 minutes with a sleep efficiency of 82.9% with the lowest oxygen saturation of 88% with periodic leg movement index 19 per hour with periodic leg movement arousal index of 1.7/hour. The patient also had actigraphy data two weeks prior to the test, and her average sleep time was six hours with multiple nap periods. The patient also stopped her Adderall (combination of amphetamine salts) and protriptyline about two weeks prior to the test. The patient underwent MSLT with CPAP use after overnight PSG with CPAP use and was found to have mean sleep latency of 1.6 minutes and five out of five sleep onset REMs. The results are mentioned below in Table 1 and Figure 1.

**Table 1:** MSLT Findings MSLT: Multiple Sleep Latency Test, REM: Rapid Eye Movement, SOREM: Sleep Onset Rapid Eye Movement

Multiple Sleep Latency Test Naps	Sleep Latency (in minutes)	Rapid Eye Movement Latency (in minutes)	Sleep Onset REM (Yes/No)
Nap 1	3.0	0.0	Yes
Nap 2	0.5	4.0	Yes
Nap 3	2.0	2.5	Yes
Nap 4	2.0	4.5	Yes
Nap 5	0.5	2.0	Yes
	Mean Sleep Latency=1.6		SOREMs=5/5

The patient also had CSF hypocretin level checked around 3 PM during the day, and it came extremely low at 50 pg/ml (abnormal if less than 110 pg/ml). This is typically seen in patients with cataplexy with narcolepsy. No diurnal variation in hypocretin level has been reported. The patient also had a genetic test done for narcolepsy, which came positive for HLA DQB1*06:02. Urine drug screen during MSLT was performed, which came negative. Therefore, the diagnosis of narcolepsy with cataplexy was made. The patient was taking Adderall before, which was stopped. The patient was put on calcium, magnesium, potassium, and sodium oxybates (Xywav) 3 gm two times during the night and solriamfetol 75 mg daily during the day. Later, pitolisant (Wakix) 35.6 mg once daily was added to her regimen, but it was changed to protriptyline 5 mg twice daily to control cataplexy due to cost issue. Her cataplexy episodes and daytime sleepiness are well controlled now. The patient also uses her CPAP to keep sleep apnea under control. The patient keeps following up in the sleep clinic, and no cataplexy episodes were observed in recent office visits.

## Discussion

Narcolepsy is characterized by the triad of excessive daytime sleepiness, hypnagogic hallucinations, and sleep paralysis. The presence of cataplexy indicates narcolepsy type I, while its absence suggests narcolepsy type II. Narcolepsy type I patients are widely associated with low CSF hypocretin levels (less than 110 pg/ml) [1].

In addition to genetic predisposition, an immunologic mediation of narcolepsy has been postulated. Streptococcal infection, H1N1 influenza infection, and influenza vaccinations have been associated with the onset of narcolepsy [5]. The immunologic pathway is either by bystander activation of autoreactive T cells or molecular mimicry. The activation of nonspecific T cells, stimulated by viral infections, can lead to the destruction of hypocretin neurons. Molecular mimicry could also be another factor in which there is activation of T cells after being presented by bacterial or viral protein particles which could also lead to the destruction of the hypocretin neurons. This method has been postulated as the cause of increased incidence of narcolepsy in the HLA DQB1*06:02 positive children after the Pandemrix vaccination in Scandinavian children [10]. Similar mechanisms have also been attributed to narcolepsy post-streptococcal infection [11]. Our patient did not have throat symptoms prior to the presentation.

Our patient was genetically susceptible to developing narcolepsy with cataplexy, with a positive HLA DQB1*06:02. Our patient also had a severely low hypocretin level at 50 pg/ml, which may lower the threshold to getting cataplexy even without an emotional trigger or minimal trigger. In addition, influenza and pneumococcal vaccines were potentially immunologic triggers that could have led to narcolepsy onset. Our patient had obesity, which may increase the risk of obstructive sleep apnea that may contribute to sleepiness and fatigue. Even after finding mild obstructive sleep apnea and treating it with CPAP in our patient, her narcolepsy symptoms did not improve. The prevalence of narcolepsy in the elderly population is exceedingly rare but has been reported in a few cases [12-14]. 

A case report describes a 69-year-old male who developed symptoms of narcolepsy type 1. He was subsequently treated with clomipramine for cataplexy and clonazepam for REM sleep behavior disorder and naps to manage daytime sleepiness [12]. Similar to our case, this patient was also positive for obstructive sleep apnea, treated with positive airway pressure. In addition, he was also positive for HLA DQB1*06:02 and had hypocretin deficiency similar to our case. However, unlike our case, there was no known potential trigger, and the patient also had REM behavior disorder.

An article published in 1987 describes two cases of delayed diagnosis of narcolepsy in elderly patients causing significant psychosocial problems [13]. Another case report describes a 60-year-old patient who presented with classic narcolepsy with frequent cataplexy. The patient had a normal MRI and CT scan. The daytime sleepiness responded to amphetamine/dextroamphetamine, and cataplexy responded to sodium oxybates [14]. This case also showed that narcolepsy with cataplexy might develop in the elderly population, but HLA typing and hypocretin measurement were not done. In addition, no potential immunologic triggers were reported, unlike our case.

Our patient received both influenza and pneumococcal vaccines, so it is hard to pinpoint which vaccine triggered the immunologic response, but it is possible with either of them.

## Conclusions

An autoimmune response, potentially provoked by influenza or pneumococcal vaccines, may lead to the development of narcolepsy with cataplexy, especially in elderly patients who are genetically susceptible. There should be a high level of suspicion that it may not need an emotional trigger to cause cataplexy if the hypocretin level is very low. If genetic testing information is available, clinicians may discuss the potential risk of developing narcolepsy about two to seven times higher in a genetically susceptible population while explaining the risks and benefits of vaccines.
